# Multi-staged gene expression profiling reveals potential genes and the critical pathways in kidney cancer

**DOI:** 10.1038/s41598-022-11143-6

**Published:** 2022-05-04

**Authors:** Hamed Ishaq Khouja, Ibraheem Mohammed Ashankyty, Leena Hussein Bajrai, P. K. Praveen Kumar, Mohammad Amjad Kamal, Ahmad Firoz, Mohammad Mobashir

**Affiliations:** 1 Department of Medical Laboratory Technology, Faculty of Applied Medical Sciences, King Abdulaziz University, Jeddah, Saudi Arabia; 2 Special Infectious Agents Unit-BSL3, King Fahad Medical Research Center, King Abdulaziz University, Jeddah, Saudi Arabia; 3 Biochemistry Department, Sciences College, King Abdulaziz University, Jeddah, Saudi Arabia; 4 Department of Biotechnology, Sri Venkateswara College of Engineering, Sriperumbudur, 602105 India; 5 West China School of Nursing/Institutes for Systems Genetics, Frontiers Science Center for Disease-Related Molecular Network, West China Hospital, Sichuan University, Chengdu, 610041 Sichuan China; 6 King Fahd Medical Research Center, King Abdulaziz University, P. O. Box 80216, Jeddah, 21589 Saudi Arabia; 7 Enzymoics, Novel Global Community Educational Foundation, 7 Peterlee Place, Hebersham, NSW 2770 Australia; 8 Department of Biological Sciences, Faculty of Science, King Abdulaziz University, Jeddah, Kingdom of Saudi Arabia; 9 SciLifeLab, Department of Oncology and Pathology, Karolinska Institutet, Box 1031, 171 21 Stockholm, Sweden

**Keywords:** Data acquisition, Data integration, Data mining, Gene ontology, Genome informatics, Microarrays, Predictive medicine

## Abstract

Cancer is among the highly complex disease and renal cell carcinoma is the sixth-leading cause of cancer death. In order to understand complex diseases such as cancer, diabetes and kidney diseases, high-throughput data are generated at large scale and it has helped in the research and diagnostic advancement. However, to unravel the meaningful information from such large datasets for comprehensive and minute understanding of cell phenotypes and disease pathophysiology remains a trivial challenge and also the molecular events leading to disease onset and progression are not well understood. With this goal, we have collected gene expression datasets from publicly available dataset which are for two different stages (I and II) for renal cell carcinoma and furthermore, the TCGA and cBioPortal database have been utilized for clinical relevance understanding. In this work, we have applied computational approach to unravel the differentially expressed genes, their networks for the enriched pathways. Based on our results, we conclude that among the most dominantly altered pathways for renal cell carcinoma, are PI3K-Akt, Foxo, endocytosis, MAPK, Tight junction, cytokine-cytokine receptor interaction pathways and the major source of alteration for these pathways are MAP3K13, CHAF1A, FDX1, ARHGAP26, ITGBL1, C10orf118, MTO1, LAMP2, STAMBP, DLC1, NSMAF, YY1, TPGS2, SCARB2, PRSS23, SYNJ1, CNPPD1, PPP2R5E. In terms of clinical significance, there are large number of differentially expressed genes which appears to be playing critical roles in survival.

## Introduction

Renal cell carcinoma (RCC) is the most common type of kidney cancer in adults, responsible for approximately 90–95% of cases and it is one of the leading causes of cancer death. Its occurrence shows mainly male predominance over women with a ratio of 1.5:1. RCC, a kidney cancer originates in the lining of the proximal convoluted tubule which is the part of the very small tubes in the kidney and transport primary urine^1,2^. High-throughput data is created at a large scale in order to understand complex diseases like cancer, and it has aided in research and diagnostic advancement^3–6^. However, extracting useful knowledge from such vast datasets for a complete and detailed understanding of cell phenotypes and disease pathophysiology remains a difficult task, and the molecular events that contribute to disease initiation and progression are still poorly understood^7–9^. The advancement of the post-genomics period has resulted in a huge amount of "big data" in biological sciences, which has led to a multitude of interdisciplinary applications in recent decades^5,10^. There are a number of biological databases that house various types of datasets. TCGA, oncomine, nephroseq, and GEO (gene expression omnibus) are the most widely used databases in biological sciences^11^. These databases mainly GEO store vast amount of datasets related with cancer, diabetes, and other biological problems^8,12–16^.

The identification of pathogenetically distinct tumour types poses a significant challenge in the treatment of complex diseases (especially cancer)^17–19^. The improvement in tumor classification always helps in the improvement during therapeutic approaches^20,21^. In target specific therapy, effectiveness can be maximised while toxicity is reduced by using enhanced classification. To access biological datasets from these databases previously, a variety of tools/approaches were used. For molecular classification of cancer Golub TR et al*.*,^22^ have divided cancer classification into two challenges as class discovery and class prediction.

A number of oncogenes and tumour suppressor genes that are changed in RCC, resulting in pathway dysregulation, need to be identified and investigated further^23–25^. Copy number, gene sequencing, expression pattern, and methylation in primary RCC are all possible avenues for achieving this goal. With continued breakthroughs in omics technology, the application of molecular markers for early diagnosis and prognosis deserves further attention^1,2,26–30^.

We have selected RCC dataset with samples from two stages (stages I and II) for the purpose of understanding how gene expression patterns vary and how altered gene expression patterns lead to possible changes in the respective inferred functions as tumour stage I to II changes and from affymetrix platforms (U133A to U133B). Different cancer stages help in describing where a cancer could be located, how far it has spread, and whether it is affecting other parts of the body^31–33^. Healthy tissue usually contains many different types of cells grouped together. If the cancer looks similar to healthy tissue and contains different cell groupings, it is called differentiated or low-grade tumor and when the cancerous tissue looks very different from the healthy tissue, it is termed as poorly differentiated or high-grade tumor. The cancer’s grade may help the clinician to predict how quickly the cancer will spread. In general, the lower the tumor’s grade, the better the prognosis. Different types of cancer have different methods to assign a cancer grade^7,34–37^. In general, it is very hard to detect most of the cancers at early stage so the main focus was on exploring the gene expression pattern alterations and its functional consequences and further to avoid biasedness, we have incorporated TCGA dataset also which have the samples from all the grades.

Here, we have selected a dataset from gene expression omnibus (GEO) where the samples are from human with two tumor stages (I and II). We have organized the samples in the order such as stage I normal versus tumor and stage II normal versus tumor for the affymetrix platforms U133A and U133B and analyzed the tumor samples with respect to their respective controls (normal sample of the same stage) for the gene expression alterations and evolved functions with the increase in tumor percentage. Based on our work, we conclude that irrespective of the tumor stage PI3K-Akt, Foxo, endocytosis, MAPK, Tight junction, cytokine-cytokine receptor interaction pathways and the major source of alteration for these pathways are MAP3K13, CHAF1A, FDX1, ARHGAP26, ITGBL1, C10orf118, MTO1, LAMP2, STAMBP, DLC1, NSMAF, YY1, TPGS2, SCARB2, PRSS23, SYNJ1, CNPPD1, PPP2R5E. In addition, we have also studied the clinical significance and observe that there are large number of differentially expressed genes which appears to be playing critical roles in for survival such as ARHGAP6, TGM4, CD248, SLC13A3, EPO, PARD6A, CLCA2, UBE2S, ERAL1, FGFR1, MRVI1, DYNC1I2, CDCA7.

## Results

**Figure 1:**
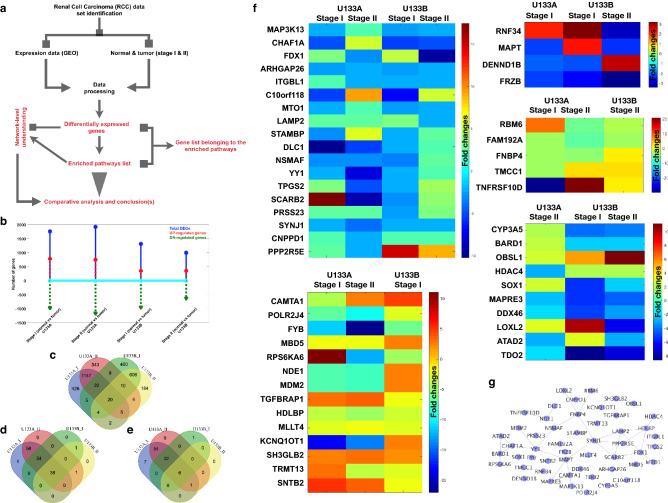
Gene expression profiling. (**a**) Workflow: from raw dataset to analysis. (**b**) Number of DEGs, up- and down-regulated genes. (**c**) Venn diagram to display the DEGs. (**d**) Venn diagram for enriched pathways for the DEGs (p-value ≤ 0.05). (**e**) Venn diagram for enriched pathways for the DEGs (p-value ≤ 0.001). (**f**) Common genes between different stages and the array chips (U133A and U133B) with their fold changes. (**g**) Mapped network for all the genes in (**f**). For venn diagram plotting, freely available webserver (http://bioinformatics.psb.ugent.be/webtools/Venn/) was used and the heatmaps and bar plot were generated by using MATLAB 2017b by using imagesc and plot commands, respectively.

In the first step, we have selected the data of our interest (raw expression dataset) GSE6344^30,38^, organized the samples in the order such as stage I normal versus tumor and stage II normal versus tumor for the affymetrix platforms U133A and U133B and processed it until normalization and log2 values for all the mapped genes as mentioned in the workflow Fig. 1a. This dataset contains 40 samples (5 normal and 5 tumor for two stages I and II from U133A and U133B platforms). For differential gene expression analysis, we have compared the tumor samples with normal samples of the respective stages and the respective platforms that it gives us four DEGs lists.

### Gene expression profiling and the associated functions for varying tumor percentages

In this study, the initial focus of our goal was to understand the gene expression pattern between the different stages for normal versus tumor samples. For this purpose, the total number of the DEGs, up, and down regulated genes have been calculated (Fig. 1b) and the number of down-regulated genes are higher than the up-regulated genes and further, we observe that the number of down regulated genes are comparatively high in all the four DEGs list (Fig. 1c). For U133A dataset, we observe very high number of DEGs for same stage and shares 1147 genes between stage I and II with respect to U133B which is 606 genes and stage I and II specific genes are also high in both the platforms U133A and U133B. Similar to the DEGs distribution, the enriched pathways are also distributed in the similar trend as shown in Fig. 1d (p-values < 0.05) and even after applying strict cut-off of p-value as shown in Fig. 1e (p-values < 0.001). Most of the shared genes between different stages and platforms have been shown with their fold changes and these genes are known to be associated with the critical pathways which are very important for multiple type of cancers (Fig. 1f). In addition, we have also mapped the known association between all these genes (from Fig. 1f) for which one list of all these DEGs have been combined to single DEGs list and finally, these genes have been mapped by using the network database in the form of network as s in Fig. 1g. Figure 1g presents the network of DEGs and their connectivity with each other where there are four smaller clusters and these clusters are connected by a core cluster of SYNJ1, MAPT, YY1, NSMAF, and FNBP4 genes and among the highly connected genes SYNJ1, LAMP2, SCARB2, FDX1, HDLBP, CHAF1A, MAPT, and FNBP4.

### Top-ranked enriched pathways for the respective DEGs list

After analyzing the number of DEGs and the enriched pathways, we have analyzed the enriched pathways and the genes which are altered in different RCC tumor stages (Table 1). We observe that MAPK, cytokine, Akt, Wnt, hippo, Hif1, metabolic signaling pathways are among the top-ranked pathways which are frequently altered and their potential source of alterations are MAP3K13, CHAF1A, FDX1, ARHGAP26, ITGBL1, C10orf118, MTO1, LAMP2, STAMBP, DLC1, NSMAF, YY1, TPGS2, SCARB2, PRSS23, SYNJ1, CNPPD1, PPP2R5E. These genes and the pathways are known to play the potential roles directly or indirectly in case of cancer.

**Table 1:** Enriched pathways grouped either common or specific to the conditions.

U133A: stage I and II U133B: stage I and II	Pl3K-Akt signaling, endocytosis, FoxO signaling, MAPK signaling, tight junction, cytokine–cytokine receptor interaction
U133A: stage I and II U133B: stage I	Neurotrophin signaling, insulin signaling, phospholipase-d signaling pathway, adipocytokine signaling, AMPK signaling, thyroid hormone synthesis, Rap1 signaling, oxytocin signaling, phagosome, apelin signaling, thyroid hormone signaling, gap junction, cGMP-PKG signaling, prolactin signaling, longevity regulating pathway, signaling pathways regulating pluripotency of stem cells, progesterone-mediated oocyte maturation, Protein processing in endoplasmic reticulum, purine metabolism, Platelet activation, axon guidance, ubiquuitin mediated proteolysis
U133A: stage I U133B: stage I and II	Ras signaling pathway
U133A: stage I U133B: stage I	Circadian entrainment, sphingolipid signaling, cell adhesion molecules (CAMs), cell cycle, TGF-beta signaling, cAMP signaling, osteoclast differentiation, TNF signaling, adrenergic signaling in card iomyocytes, peroxisome, T cell receptor signaling, hematopoietic cell lineage, natural killer cell mediated cytotoxicity, Fc gamma R-mediated phagocytosis, HIF-1 signaling, oocyte meiosis, NF-kappa B signaling, leukocyte transendothelial migration, Fc epsilon Rl signaling, valine leucine and isoleucine degradation, ECM-receptor interaction, phosphatidylinositol signaling system, estrogen signaling, ErbB signaling, pyrimidine metabolism, long-term potentiation, Regulation of actin cytoskeleton, retrograde endocannabinoid signaling, vascular smooth muscle contraction, inflammatory mediator regulation of TRP channels, RNA transport, apoptosis, Focal adhesion, notch signaling, renin secretion, Jak-STAT signaling, melanogenesis, calcium signaling, VEGF signaling, B cell receptor signaling, oxidative phosphorylation, Spliceosome, long-term depression, drug metabolism—cytochrome P450, glycerophospholipid metabolism, neuroactive ligand-receptor interaction, tryptophan metabolism, p53 signaling pathway, Antigen processing and presentation, Wnt signaling, Hippo signaling, toll-like receptor signaling, GnRH signaling pathway, adherens junction
U133A: Stage I	Olfactory transduction, PPAR signaling, inositol phosphate metabolism, RIG-I-like receptor signaling, lysine degradation, taste transduction, ovarian steroidogenesis
U133B: Stage I	Butanoate metabol ism, synaptic vesicle cycle, tyrosine metabolism, drug metabolism-other enzymes, mRNA surveillance pathway, steroid hormone biosynthesis, proteasome, metabolism of xenobiotics by cytochrome P450, retinol metabolism
U133A: Stage II	Ribosome

These pathways have been generated after plotting the venn diagram.

### Network-level understanding of the DEGs

**Figure 2:**
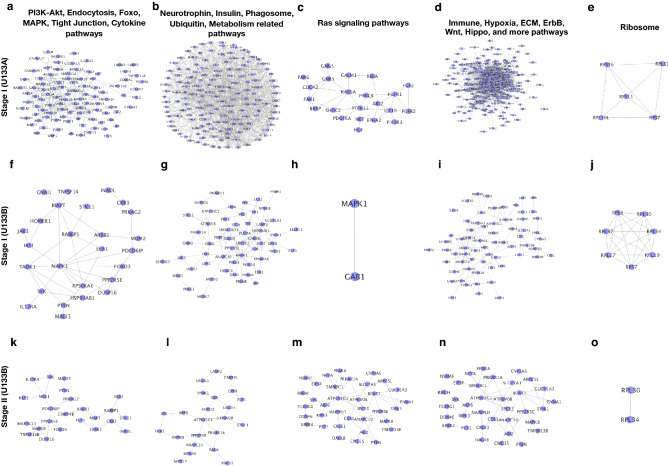
Networks for the genes matched with those pathways which are enriched (p-values ≤ 0.001) and common shown in venn diagram for all the four DEGs list (Stage I and II for U133A and U1333B). In this figure, we have selected those pathways which are commonly enriched pathways and mapped the genes belonging to these pathways from the DEGs list and finally mapped out the networks. (**a**–**e**) It represents the networks for Stage I of U133A platform data for the list of pathways. (**f**–**j**) It represents the networks for Stage I of U133B platform data for the list of pathways. (**k**–**o**) It represents the networks for Stage II of U133B platform data for the list of pathways. All these networks, were drawn by using cytoscape software.

**Figure 3:**
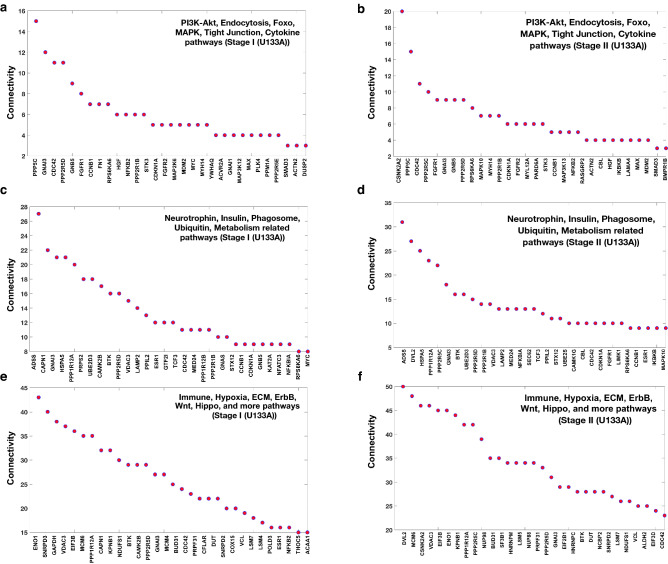
Connectivity in the selected networks (where the gene connectivity is not visible), for the top 30 genes matched with those pathways which are enriched for the DEGs list. (**a**–**f**) Connectivity of the genes in the network for Stage I and II of U133A. These sub-figures were plotted by using MATLAB 2017b by using plot command and afterward dot option was selected and the line was removed.

Based on the venn diagram of the enriched pathways, we have prepared the list of the pathways in five groups (commonly enriched) and matched the genes with these pathways lists from all the four DEGs list (normal versus tumor in stage I and II for the U133A and U133B datasets). In Fig. 2, the networks have been shown for stage I of U133A, Stage I and II of U133B datasets. The networks shown are for those DEGs which are matching to different pathways lists obtained during venn diagram drawing. The major pathways have been highlighted on the top of the figure and in the left side the tumor stage have been mentioned. Since most of the networks for stage II of U133A dataset were densely connected so for such networks we have presented top 30 genes in terms of connectivity within the network (Fig. 3). Here, we have also shown the connectivity of the genes for those networks where the connections are not clearly visible. For more details of the list of the genes and the pathways used for the network-level analysis were supplied in the Supplementary Table S1.

### Clinical significance of the differentially expressed genes

**Figure 4:**
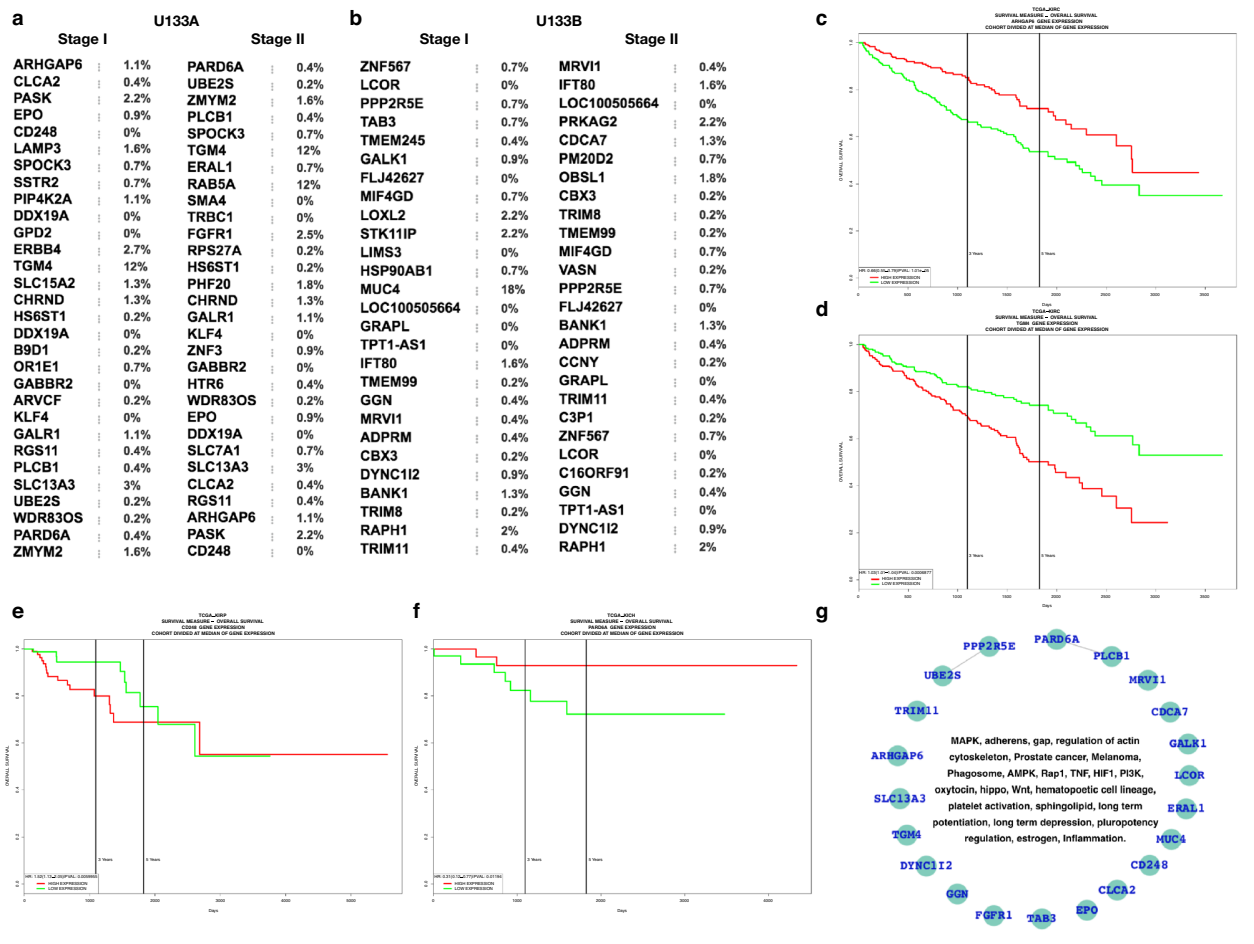
Clinical significance of the top ranked genes. (**a**,**b**) Top 30 (15 up and down) DEGs (based on fold change) with the rate of mutation in kidney renal clear cell carcinoma (TCGA) with their mutations. (**c**–**f**) Survival plots for the selected top ranked genes. (**g**) Network of the clinically significant genes and the associated pathways. Here, (**a**) and (**b**) were drawn by using cBioPortal, (**c**–**f**) by using PROGgeneV2 (http://www.progtools.net/gene/index.php), and (**g**) by cytoscape.

**Figure 5:**
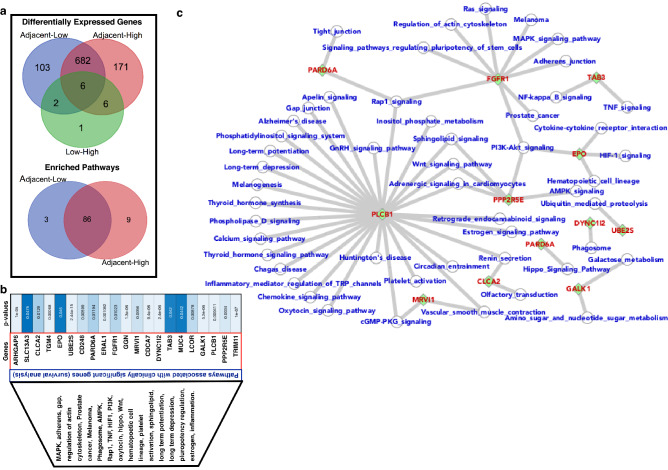
Cross-analysis and clinical relevance. (**a**) Expression profiling of the genes for early (low grade) and advanced stages (high grade) and the tumors with adjacent normal tissues. (**b**) Survival analysis. Genes appear to be clinically significant in terms of survival analysis and the critical pathways associated with them and (**c**) their associations and to draw such association, the KEGG database was used. The green color and diamond shape node represent the gene and the circular node represent the pathway^59–61,71^.

Additionally, we have selected the top-ranked genes (based on the fold change 15 up and 15 down) and analyzed the patients survival (Kaplan–Meier plot) for the patient samples from TCGA database and the dataset was TCGA kidney renal clear cell carcinoma (source data from GDAC Firehose) which contains 538 samples^5,36,39^. We observe that most of the top-raked genes (from selected 30 DEGs) mainly up-regulated genes show very high significance on the patients survival (Fig. 4). In this figure, we have also shown the mutations in these top-ranked DEGs for clear renal cell carcinoma in the TCGA databse. There are few genes(ERBB4, SLC13A3, TGM4, and FGFR1) which are mutated at very high rate as shown in Fig. 4a,b. Further, we have also selected different dataset (GSE68417^40^) which contains the samples for adjacent normal, low grade, and high grade and compared the differentially expressed genes and the enriched pathways with each other (Fig. 5a). This shows that the DEGs of adjacent normal versus low grade tumor samples share majority of the DEGs of adjacent normal versus high grade tumor samples and both these list share few DEGs with low grade versus high grade DEGs list and as expected there was no shared enriched pathways at all because there appears only few genes which have gene expression with fold change ≥  + 1.5 (up regulated) or ≤ − 1.5 (down regulated) in case of low grade versus high grade. Kaplan–Meier plots show the clinical significance and that is a large number of differentially expressed genes appear to be potentially significant in terms of survival and some of the selected genes are ARHGAP6, TGM4, CD248, SLC13A3, EPO, PARD6A, CLCA2, UBE2S, ERAL1, FGFR1, MRVI1, DYNC1I2, CDCA7 (additional data shown in supplementary Figs. S1–S6). Moreover, Fig. 5b has been presented with the list of genes and the respective p-values for survival analysis and here only those genes have been shown which are clinically significant and the overall pathways associated with these genes and further specific assocations were shown in Fig. 5c. Additionally, the expressions (RNA and protein) have been shown in supplementary data S7. We have checked the expression of these clinically relevant genes by using protein atlas where most of these genes are expressed in case of RCC and act as biomarkers and only TGM4 and GGN were not expressed.

## Discussion

Renal cell carcinoma is one of the most common cancers, and it is one of the leading causes of cancer death^14,15,41^. In terms of therapy and diagnosis, therapeutic and clinical outcomes differ between the individuals with even close similarity in clinical and pathological characteristics (tumor type, grades, and stages) and despite tremendous efforts to identify molecular biomarkers (prognostic and predictive) and with improved precision compared to clinical and pathological predictors only few molecular tests have been introduced into oncological practice^29^. So it is important to understand and unravel different levels (such as gene expression pattern, epigenetics, protein expression) of diversities in cancer^42,43^. We gathered the previously published dataset for this purpose and conducted a detailed and precise study ranging from gene expression profiling to functional changes, including networks mapped from the human protein network database.

Our work leads to the conclusion that irrespective of the tumor stage PI3K-Akt, Foxo, endocytosis, MAPK, Tight junction, cytokine-cytokine receptor interaction pathways and the major source of alteration for these pathways are MAP3K13, CHAF1A, FDX1, ARHGAP26, ITGBL1, C10orf118, MTO1, LAMP2, STAMBP, DLC1, NSMAF, YY1, TPGS2, SCARB2, PRSS23, SYNJ1, CNPPD1, PPP2R5E. Networks of DEGs for the enriched pathways show that there are large number of genes from few specific pathways are altered such as Ras signaling pathways(Fig. 2c,h,m), immune sysytems, Wnt, hippo, (Fig. 2d,i,n) Akt pathways (Fig. 2a,f,k). Here, we observe that critical pathways altered in RCC are wnt, hippo, regulation of actin cytoskeleton, ECM, infection and inflammation, metabolic, and more cancer related pathways. From the mapped network, we observe that the highly connected genes infer the potential pathways or in other works the top ranked genes based on connectivity refer to those pathways which are directly or indirectly associated either with RCC or other types of cancer.

In terms of clinical significance, we looked at the rate of mutations for the top ranked genes (based on fold change) and patients' survival for changes in gene expression, with Kaplan–Meier plots indicating clinical significance. We conclude that a large number of differentially expressed genes tend to be potentially important in terms of survival, with ARHGAP6, TGM4, CD248, SLC13A3, EPO, PARD6A, CLCA2, UBE2S, ERAL1, FGFR1, MRVI1, DYNC1I2, CDCA7 among the genes chosen. Using the publicly available datasets, we have investigated the gene expression profiling for renal cell carcinoma. In the previous work, it has been focused on selected genes and pathways. Here, we have investigated the list of critical pathways and the genes which appear to be clinically highly significant in case of renal cell carcinoma. These clinically significant genes lead to potential alteration in PI3K-Akt, foxo, endocytosis, MAPK, tight junction, cytokine-cytokine receptor interaction pathways. Our work will help in diagnosing the renal cell carcinoma patients because here, we have presented the differentially expressed genes, their inferred pathways, and the clinical impact of the selective genes. Since, our finding is from overall perspective including clinical relevance so this study will help in future for diagnostic also.

This work also appears to be more unique in comparison to the previous study that we potentially explored grade I and II of RCC and further explored the clinical relevance. Healthy tissue usually contains many different types of cells grouped together and if the cancer looks similar to healthy tissue and contains different cell groupings, it is called differentiated or low-grade tumor and when the cancerous tissue looks very different from the healthy tissue, it is termed as poorly differentiated or high-grade tumor. The cancer’s grade may help the clinician to predict how quickly the cancer will spread. In general, the lower the tumor’s grade, the better the prognosis. Different types of cancer have different methods to assign a cancer grade^7,34–37^ and the different tumor stages could help in describing the severeness, tumor propagation speed, and its impact on the other organs^31–33^.. In general, it is very hard to detect most of the cancers at early stage so the main focus was on exploring the gene expression pattern alterations and its functional consequences and further to avoid biasedness, we have incorporated TCGA dataset also which have the samples from all the grades. Further, we have also investigated the expression of these clinically relevant genes by using protein atlas (https://www.proteinatlas.org/)^44–48^. We observe that most of these genes are expressed in case of RCC and act as biomarkers and only TGM4 and GGN were not expressed. This study will be an important step for the understanding of early stage tumor propagation and also will be helpful for clinical aspect.

## Conclusions

Based on our findings, we conclude that PI3K-Akt, Foxo, endocytosis, MAPK, Tight junction, and cytokine-cytokine receptor interaction pathways are among the most commonly altered pathways in renal cell carcinoma, and that MAP3K13, CHAF1A, FDX1, ARHGAP26, ITGBL1, C10orf118, MTO1, LAMP2, STAMBP, DLC1, NSMAF, YY1, TPGS2, SCARB2, PRSS23, SYNJ1, CNPPD1, and PPP2R5E are the major sources of alteration for these pathways. Wnt, hippo, actin cytoskeleton control, ECM, infection and inflammation, metabolic, and other cancer-related pathways are among the most important pathways altered in RCC. ARHGAP6, TGM4, CD248, SLC13A3, EPO, PARD6A, CLCA2, UBE2S, ERAL1, FGFR1, MRVI1, DYNC1I2, CDCA7 are some of the genes that were chosen after survival study.

## Methods

Here, GSE6344 dataset was used for the study which contains the samples of stage I and II of gene expression for tumor kidney cancer^30,38^. In the first step, we selected the raw expression dataset GSE6344 and processed it until normalisation and log2 values of all mapped genes were achieved, as shown in Fig. 1a of the workflow. These 40 samples in this dataset were 5 normal and 5 tumor for two stages I and II from U133A and U133B platforms. We have compared the tumor samples with standard samples of the respective stages and platforms for differential gene expression analysis, yielding four DEGs lists.

In short the basic steps involved for the entire study are raw file processing, intensity calculation and normalization. For normalization^49–51^, GCRMA^52–56^, RMA, and EB are the most commonly used approaches. Here, we have used EB for raw intensity normalization. After normalization, we proceed for our goal which is to understand the gene expression patterns^14,57^ and its inferred functions^57,58^.

To prepare the list of DEGs and analysis, we have our own in-built codes. The samples were placed into two groups such as COVID-19 positive and negative and then normal and the tumor samples. The selection criteria were placed by the fold change and p-values which have been calculated and for the selection of genes as differentially expressed the threshold of fold changes and p-values applied were ± 2 and 0.05, respectively and then KEGG database^59–61^ have been used for pathway analysis and for which there is our own code designed^62^. In summary, for differential gene expression prediction and statistical analysis, MATLAB2017 functions (e.g., mattest) were applied and further for pathway analysis, we used KEGG^61^ database^62–65^.

For generating DEGs network, FunCoup2.0^66^ has been used for all the networks throughout the work and cytoscape^67^ has been used for network visualization. For most of our coding and calculations MATLAB has been used^62–65^. Furthermore, FunCoup2.0^66^ database and cytoscape and its applications^68^ were used for network visualization to understand the network and the connectivity of the genes within the network of DEGs^69,70^. The basic concept of FunCoup network database is that it predicts four different classes of functional coupling or associations such as protein complexes, protein–protein physical interactions, metabolic, and signaling pathways^66^. MATLAB 2017b codes and the command line codes have been used for figure plotting and during analysis. For the network level-analysis such as the number of connectivity per gene and the genes belonging to different number of pathways, the codes have been written in MATLAB and finally it has been plotted also by the codes written in MATLAB^64,65^. For venn diagram plotting, freely available webserver (http://bioinformatics.psb.ugent.be/webtools/Venn/) was used^72–74^.

## Supplementary Information

Supplementary Figures.

Supplementary Table S1.

## Data Availability

We have utilized the publicly available datasets (main data source) which are freely available and have mentioned it in method section with proper references. The analyzed details have been supported by the supplementary data.
