# Supplementary material for: Multi-staged gene expression profiling reveals potential genes and the critical pathways in kidney cancer
Source: Sci Rep. 2022 May 4;12:7240. doi: 10.1038/s41598-022-11143-6 (PMC9065671; doi:10.1038/s41598-022-11143-6)

S1

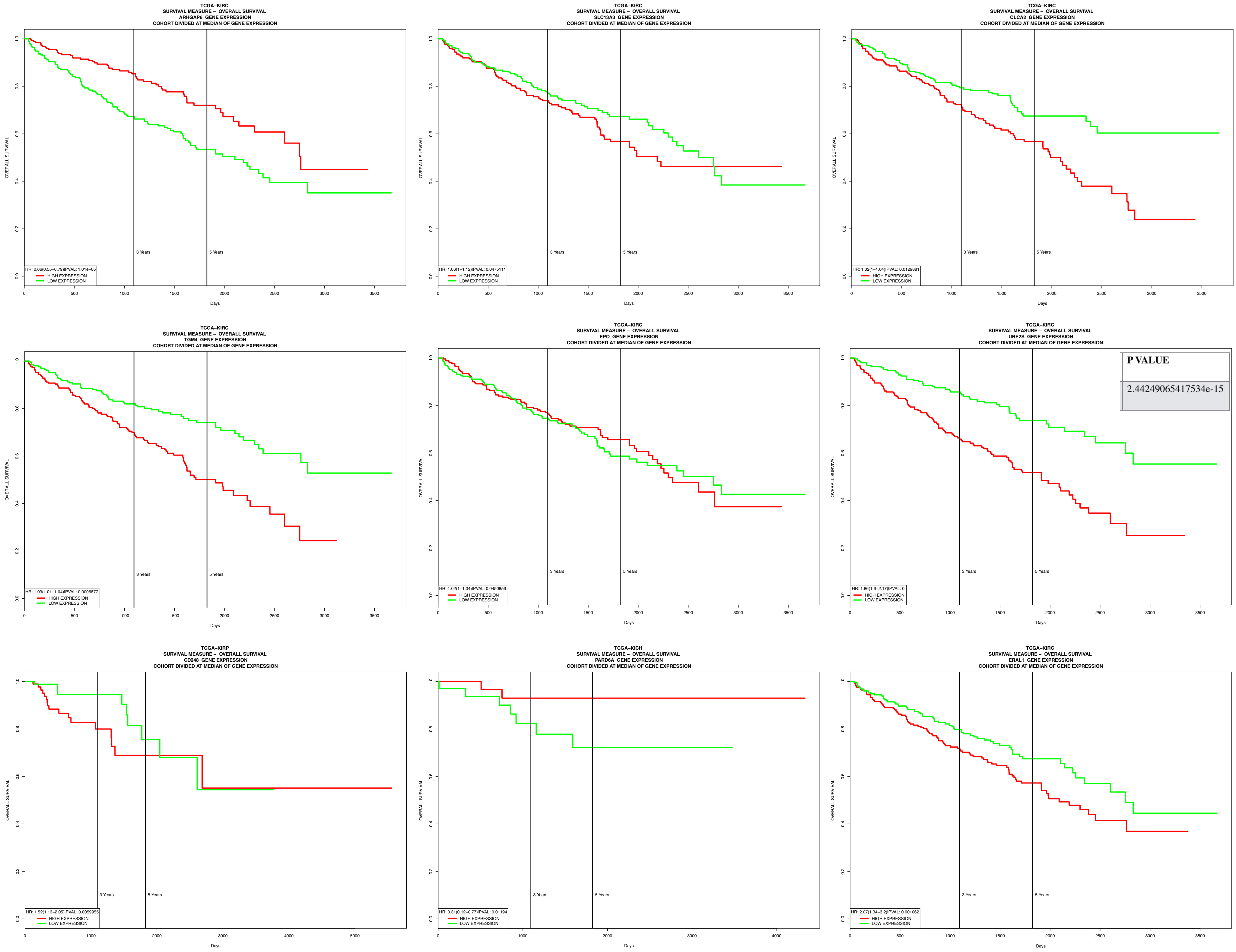
TCGA–KIRC
SURVIVAL MEASURE – OVERALL SURVIVAL
ARHGAP6 GENE EXPRESSION
COHORT DIVIDED AT MEDIAN OF GENE EXPRESSION
HR: 0.66(0.55–0.79)IPVAL: 1.01e–05
HIGH EXPRESSION
LOW EXPRESSION
TCGA–KIRC
SURVIVAL MEASURE – OVERALL SURVIVAL
SLC13A3 GENE EXPRESSION
COHORT DIVIDED AT MEDIAN OF GENE EXPRESSION
HR: 1.06(1–1.12)IPVAL: 0.0475111
HIGH EXPRESSION
LOW EXPRESSION
TCGA–KIRC
SURVIVAL MEASURE – OVERALL SURVIVAL
CLCA2 GENE EXPRESSION
COHORT DIVIDED AT MEDIAN OF GENE EXPRESSION
HR: 1.02(1–1.04)IPVAL: 0.0129881
HIGH EXPRESSION
LOW EXPRESSION
TCGA–KIRC
SURVIVAL MEASURE – OVERALL SURVIVAL
TGM4 GENE EXPRESSION
COHORT DIVIDED AT MEDIAN OF GENE EXPRESSION
HR: 1.03(1.01–1.04)IPVAL: 0.0006877
HIGH EXPRESSION
LOW EXPRESSION
TCGA–KIRC
SURVIVAL MEASURE – OVERALL SURVIVAL
EPO GENE EXPRESSION
COHORT DIVIDED AT MEDIAN OF GENE EXPRESSION
HR: 1.02(1–1.04)IPVAL: 0.0450856
HIGH EXPRESSION
LOW EXPRESSION
TCGA–KIRC
SURVIVAL MEASURE – OVERALL SURVIVAL
UBE2S GENE EXPRESSION
COHORT DIVIDED AT MEDIAN OF GENE EXPRESSION
P VALUE
2.44249065417534e-15
HR: 1.86(1.6–2.17)IPVAL: 0
HIGH EXPRESSION
LOW EXPRESSION
TCGA–KIRP
SURVIVAL MEASURE – OVERALL SURVIVAL
CD248 GENE EXPRESSION
COHORT DIVIDED AT MEDIAN OF GENE EXPRESSION
HR: 1.52(1.13–2.05)IPVAL: 0.0059955
HIGH EXPRESSION
LOW EXPRESSION
TCGA–KICH
SURVIVAL MEASURE – OVERALL SURVIVAL
PARD6A GENE EXPRESSION
COHORT DIVIDED AT MEDIAN OF GENE EXPRESSION
HR: 0.31(0.12–0.77)IPVAL: 0.01194
HIGH EXPRESSION
LOW EXPRESSION
TCGA–KIRC
SURVIVAL MEASURE – OVERALL SURVIVAL
ERAL1 GENE EXPRESSION
COHORT DIVIDED AT MEDIAN OF GENE EXPRESSION
HR: 2.07(1.34–3.2)IPVAL: 0.001062
HIGH EXPRESSION
LOW EXPRESSION
OVERALL SURVIVAL
Days
3 Years
5 Years

S2

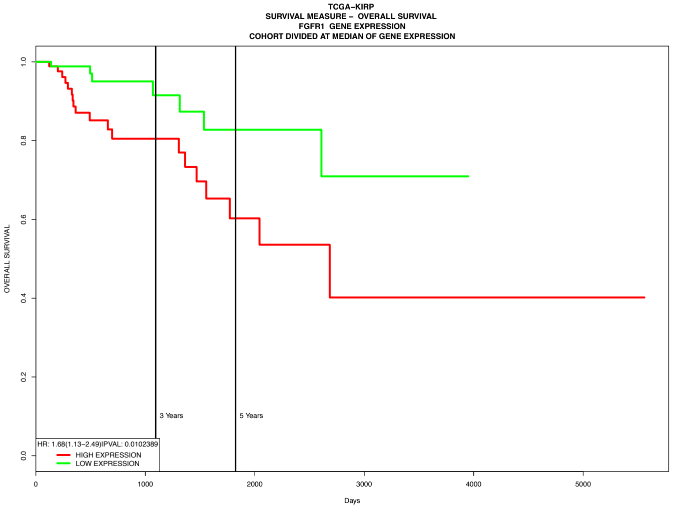

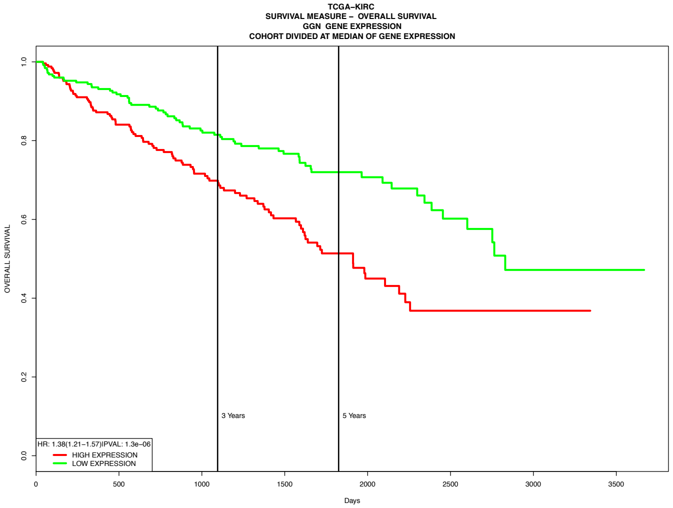

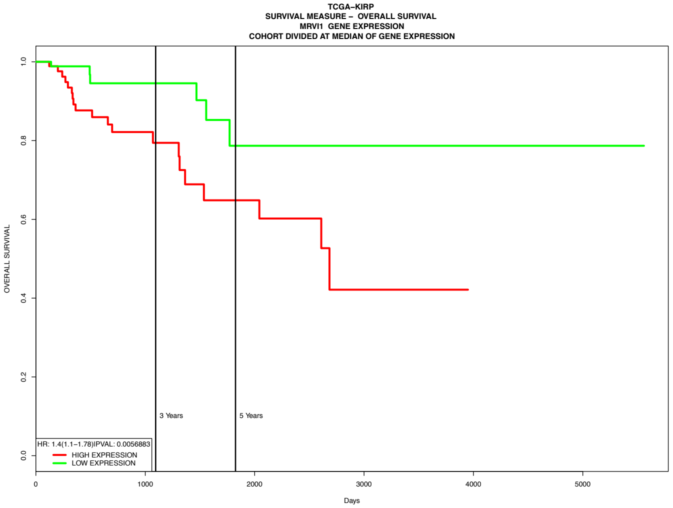

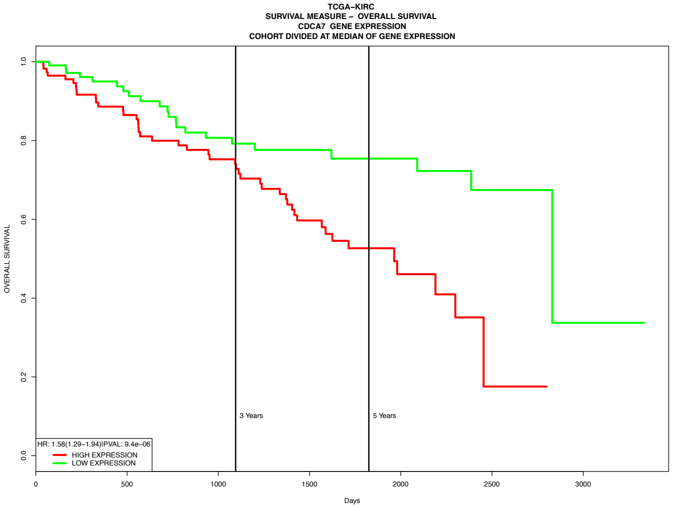

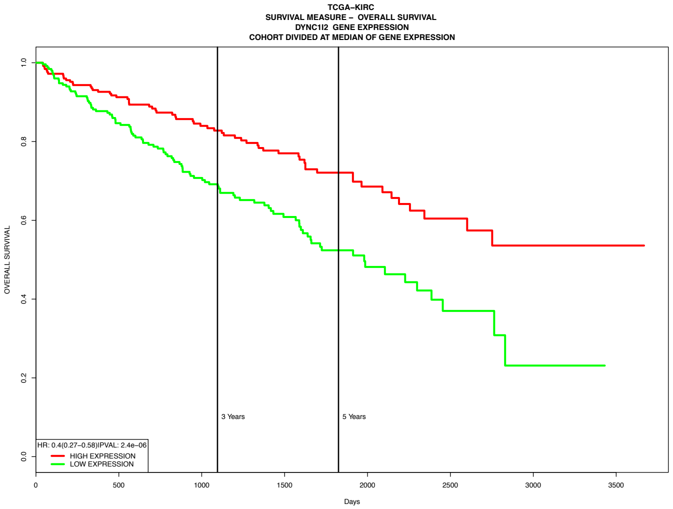

S1—S2. Survival analysis of the top-ranked genes based on our analysis. Here, only those plots have been shown which are clinically highly significant (p-value < 0.05).

S3

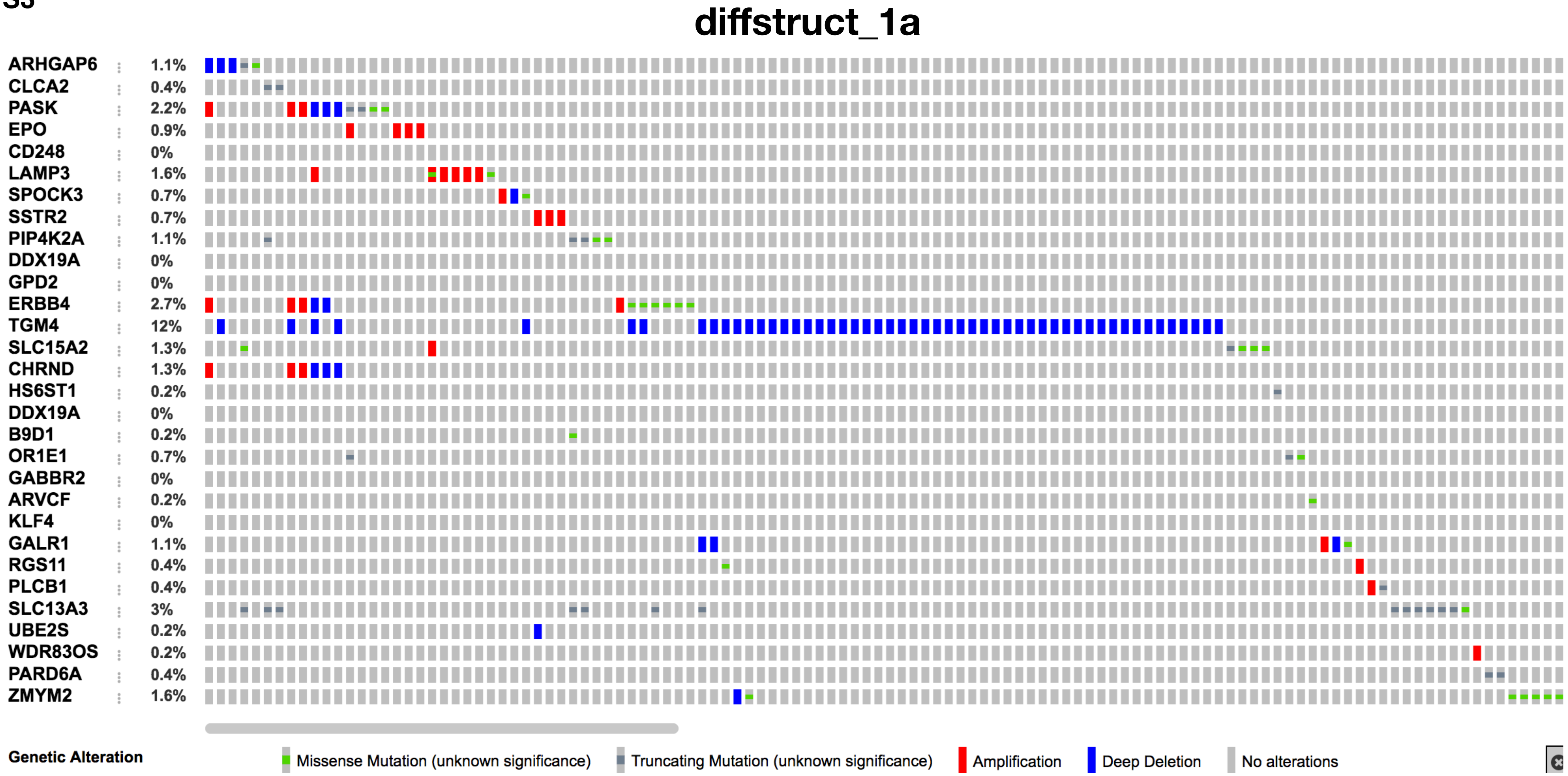

S3—S6. Top-ranked genes which were further analyzed by using cBioPortal and TCGA database in case of kidney cancer. The top-ranked genes have been processed for further analysis in terms of genetic alteration in clinical samples.

S4

diffstruct_1b

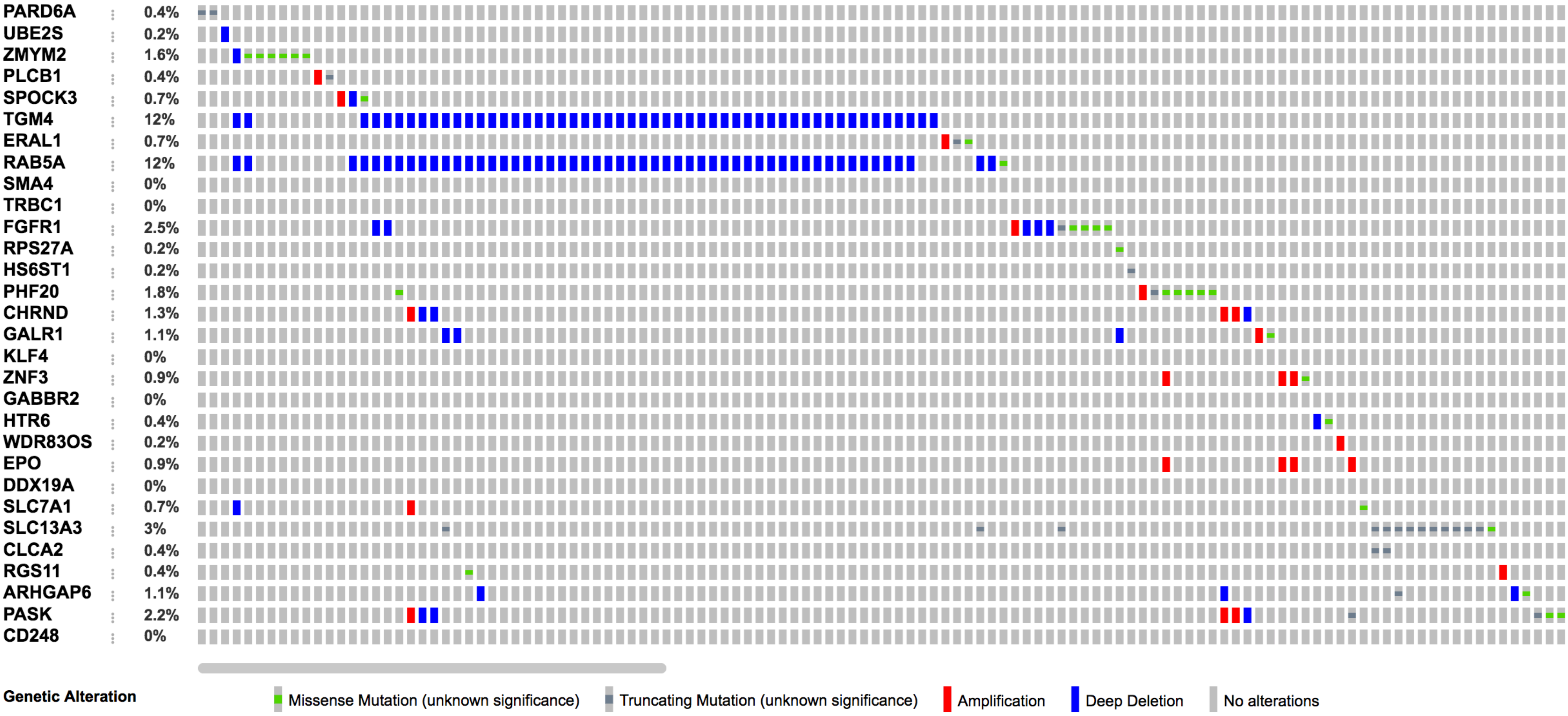

S5

diffstruct_2a

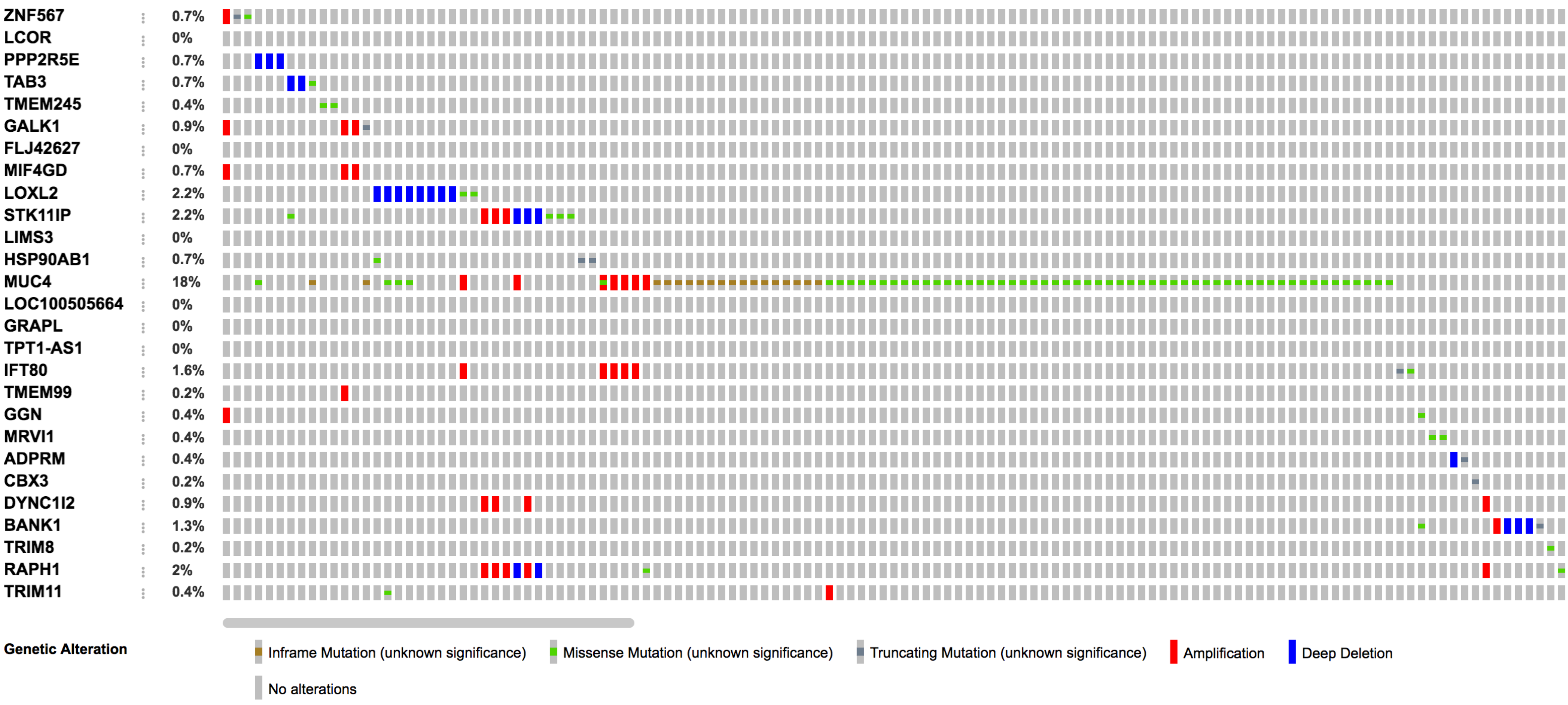

S6

diffstruct_2b

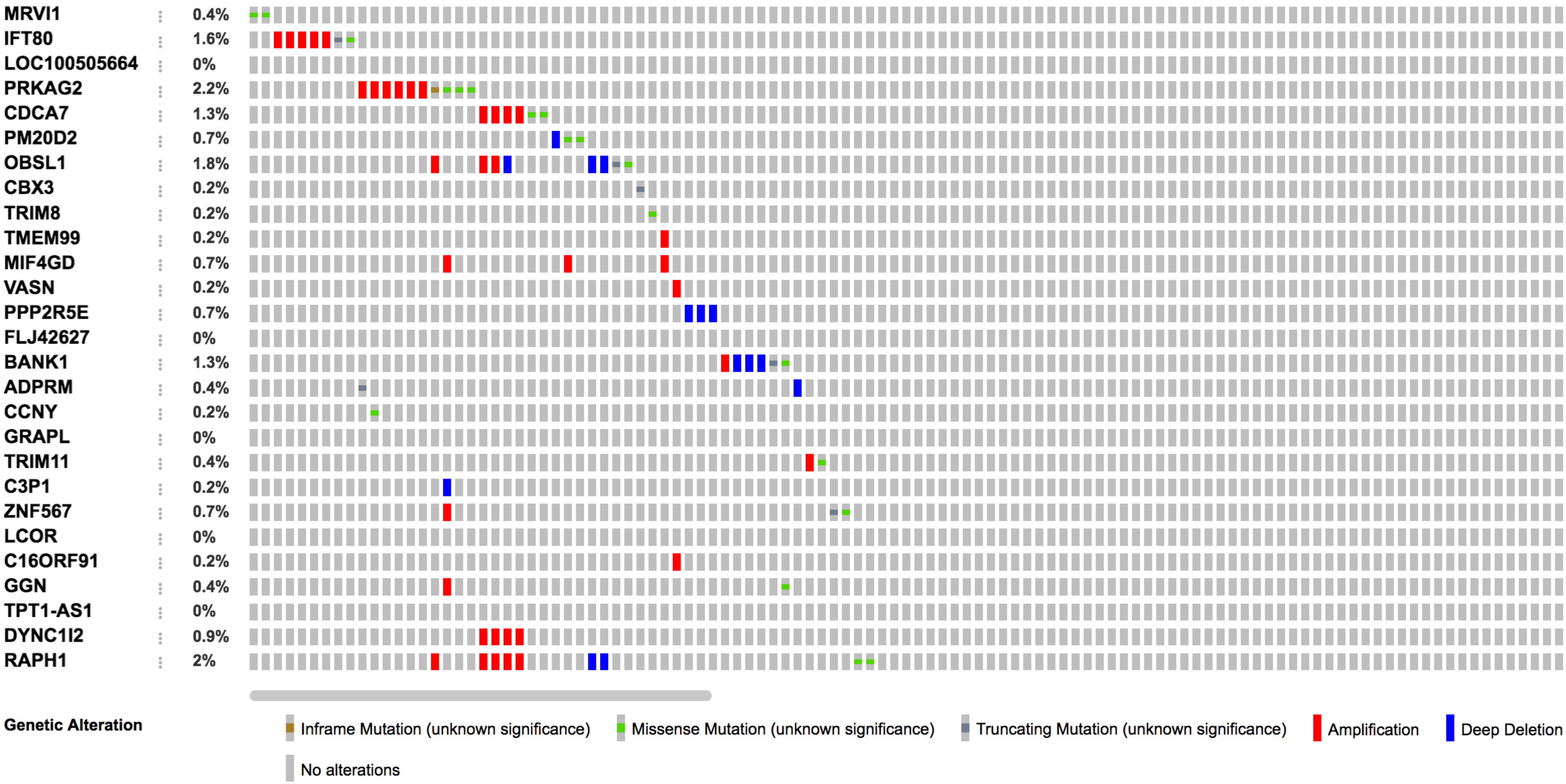

S7

Expression of clinically relevant genes in case of RCC (source protein atlas)

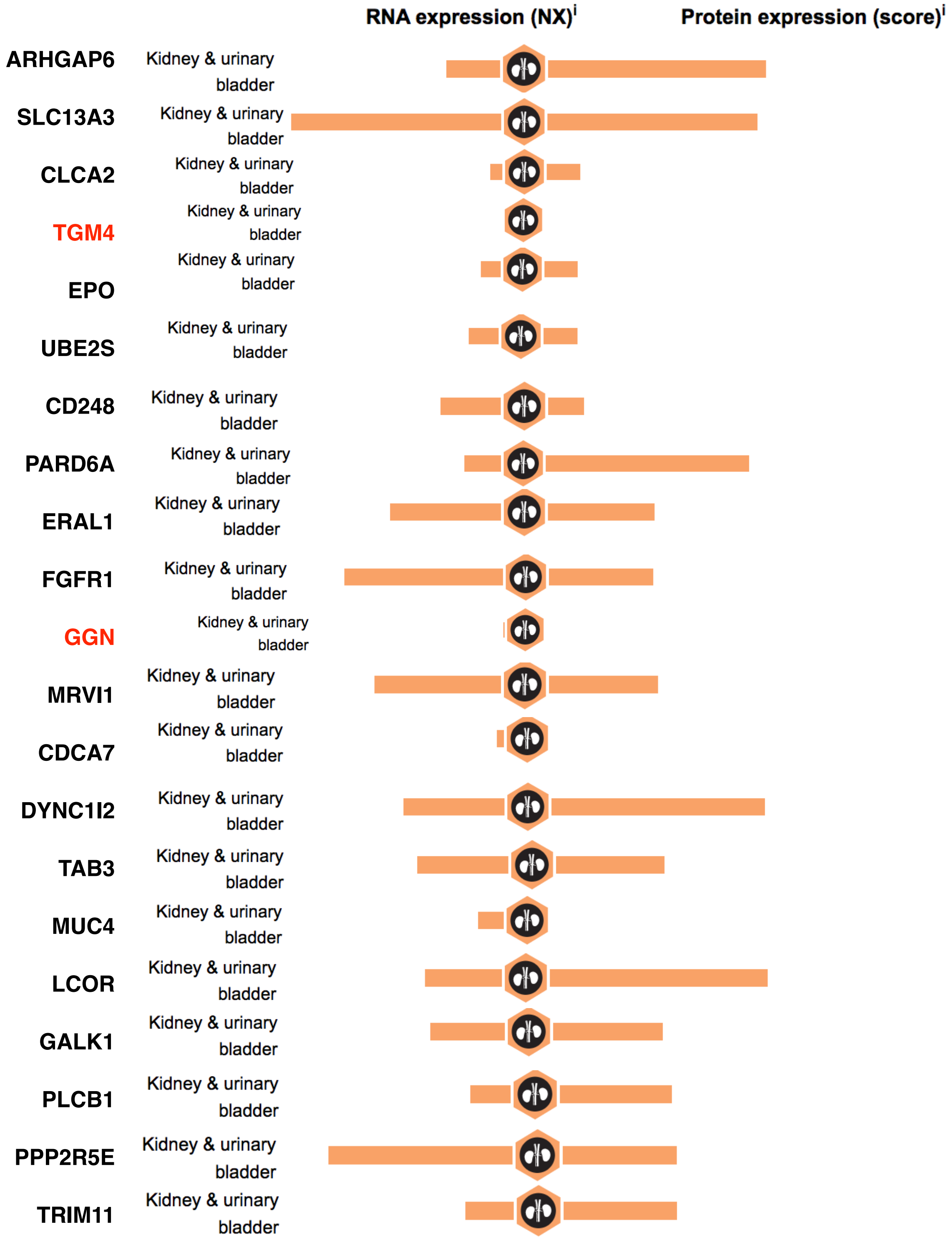

Supplement: Supplementary file 1 — Supplementary Figures. [file 41598_2022_11143_MOESM1_ESM.pdf]
